# Dendritic Cells Are the Intriguing Players in the Puzzle of Idiopathic Pulmonary Fibrosis Pathogenesis

**DOI:** 10.3389/fimmu.2021.664109

**Published:** 2021-04-30

**Authors:** Marialuisa Bocchino, Serena Zanotta, Ludovica Capitelli, Domenico Galati

**Affiliations:** ^1^ Respiratory Medicine Division, Department of Clinical Medicine and Surgery, Federico II University, Naples, Italy; ^2^ Hematology−Oncology and Stem Cell Transplantation Unit, Department of Hematology and Developmental Therapeutics, Istituto Nazionale Tumori-IRCCS-Fondazione G. Pascale, Naples, Italy

**Keywords:** idiopathic pulmonary fibrosis, dendritic cells, immunity, cancer, immunotherapy

## Abstract

Idiopathic pulmonary fibrosis (IPF) is the most devastating progressive interstitial lung disease that remains refractory to treatment. Pathogenesis of IPF relies on the aberrant cross-talk between injured alveolar cells and myofibroblasts, which ultimately leads to an aberrant fibrous reaction. The contribution of the immune system to IPF remains not fully explored. Recent evidence suggests that both innate and adaptive immune responses may participate in the fibrotic process. Dendritic cells (DCs) are the most potent professional antigen-presenting cells that bridge innate and adaptive immunity. Also, they exert a crucial role in the immune surveillance of the lung, where they are strategically placed in the airway epithelium and interstitium. Immature DCs accumulate in the IPF lung close to areas of epithelial hyperplasia and fibrosis. Conversely, mature DCs are concentrated in well-organized lymphoid follicles along with T and B cells and bronchoalveolar lavage of IPF patients. We have recently shown that all sub-types of peripheral blood DCs (including conventional and plasmacytoid DCs) are severely depleted in therapy naïve IPF patients. Also, the low frequency of conventional CD1c^+^ DCs is predictive of a worse prognosis. The purpose of this mini-review is to focus on the main evidence on DC involvement in IPF pathogenesis. Unanswered questions and opportunities for future research ranging from a better understanding of their contribution to diagnosis and prognosis to personalized DC-based therapies will be explored.

## Introduction

Idiopathic pulmonary fibrosis (IPF) is a progressive and devastating fatal lung disease that usually remains refractory to treatment (1–3), with an estimated median survival of 2 to 5 years from the first diagnosis. In the last two decades, disease incidence has steadily increased, varying from 2.8 to 19 cases per 100 .000 people per year in Europe and North America, respectively (1). Disease behavior is also highly variable, with associated comorbidities potentially exerting a detrimental impact on prognosis (4, 5). The current availability of anti-fibrotic drugs (i.e., nintedanib and pirfenidone) has improved patients’ short-term life expectancy through the slowdown of the lung function decline and the reduction of hospitalization rate and episodes of acute exacerbation (6). Despite many efforts, the pathogenesis of IPF has not yet been elucidated. No longer considered just an inflammatory disorder (7), IPF pathogenesis likely relies on the aberrant cross-talk between injured alveolar cells and myofibroblasts. This interaction ultimately promotes a pro-fibrotic microenvironment through the engagement of a vicious circle supported, among others, by oxidative stress (8–10). The immune system’s contribution to IPF remains poorly understood, with several pieces of emerging evidence suggesting that both innate and adaptive responses can orchestrate the fibrotic process (11–13). In this scenario, dendritic cells (DCs) may play a significant role because of their involvement in the lungs’ immune surveillance, where they are strategically placed within the airway epithelium and interstitium (14).

Notably, DCs encompass a heterogeneous family of bone marrow-derived cells recognized as the most specialized and potent antigen-presenting cells (APCs) of the immune system (15, 16). DCs are located in almost all tissues, where they detect and process Ags for presentation to T lymphocytes, thus establishing a tailored link between innate and adaptive immune responses. Besides, DCs are pivotal in regulating the delicate interplay between immunity and tolerance (17–19) as they promote the deletion of clonal autoreactive immature T cells in the thymus. Conversely, DCs interact in the periphery with T cells to achieve immune tolerance by inducing T-cell anergy, T cell deletion, and amplification and stimulation of regulatory T cell (Treg) subsets (18, 19). Due to their pleiotropic functions and properties within the immune system, DCs have been broadly studied in different experimental and internal medicine areas, including transplantation, allergy, autoimmunity, infectious diseases, cancer (20), and, more recently, fibrosis. Significant efforts have explored the fibrogenesis of different organs, including the liver, the kidney, and the heart (21–24).

The present review aims to offer an overview including the most relevant contributions in the field of IPF to focus on the emerging evidence addressing the role of DCs in disease pathogenesis and clinical behavior and potentially in immune-targeted therapy development.

## Development of Dendritic Cells

**Figure 1:**
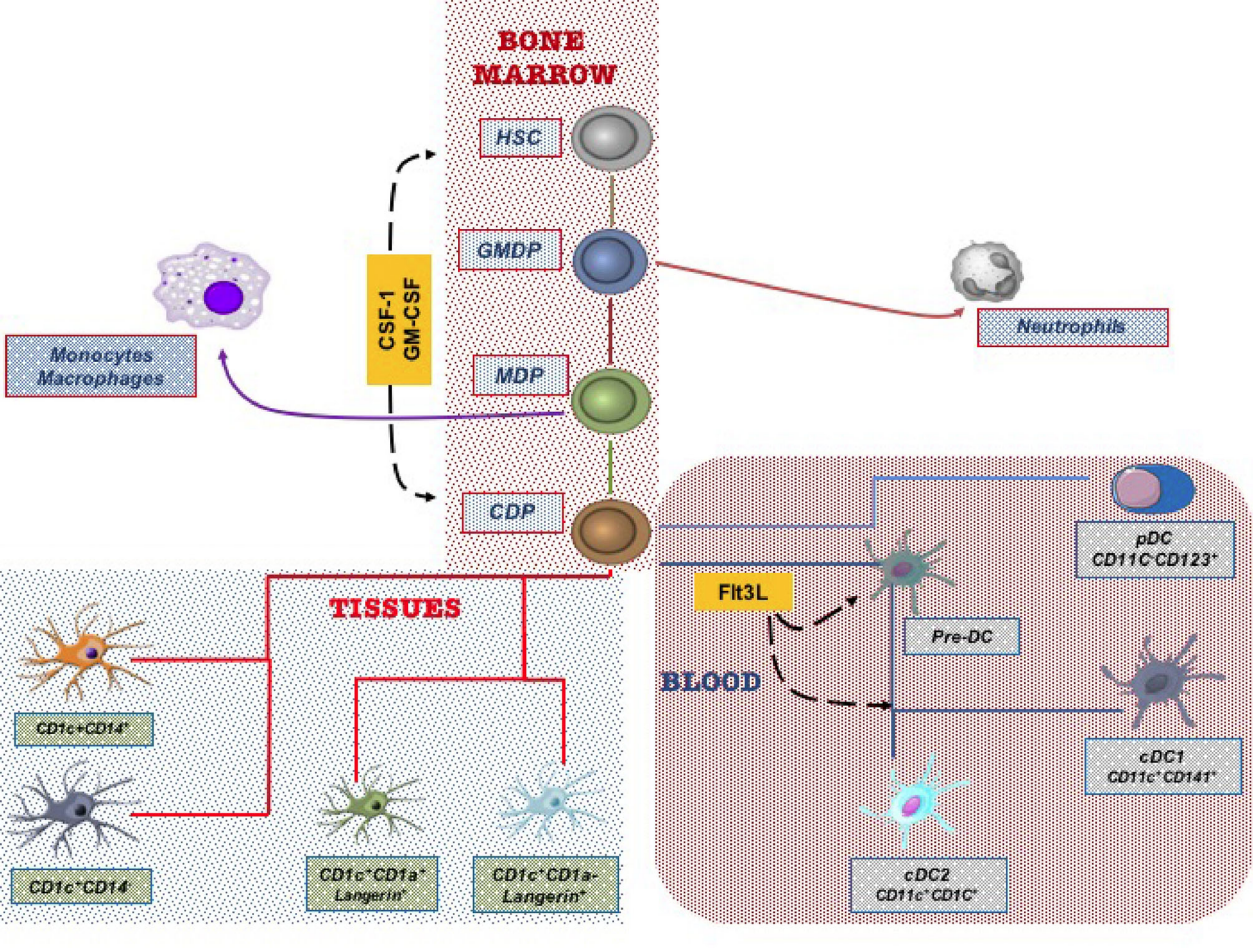
Dendritic cells (DCs) derive from hematopoietic stem cells in the bone marrow. Progenitor cells give rise in the final step to Common Derived Progenitors (CDPs) that differentiate in the blood circulating DC subtypes and in lung tissue DC subsets.

DCs originate from bone marrow progenitors through hematopoiesis, a finely regulated development process that involves several cellular and molecular events. Recent studies have identified a common DC precursor, the human granulocyte-monocyte-DC progenitor (GMDP), which supports the development of all the three major human DC subtypes (25). The GMDPs, through an intermediate maturation state into monocyte-dendritic progenitors (MDPs), differentiate into the common DC progenitors (CDPs). CDPs are restricted to the bone marrow, where they give rise to plasmacytoid DCs (pDCs) and conventional DC precursors (pre-cDCs). Frequencies of pre-cDCs increase in response to circulating FMS-like tyrosine kinase-3 Ligand (Flt3L) and then terminally differentiate into conventional DC (cDC) subsets in the periphery (25, 26). Accordingly, colony-stimulating factor-1 (CSF-1) and granulocyte-macrophage colony-stimulating factor (GM-CSF) are major cytokines required for human DC differentiation. In particular, Flt3L is a crucial regulator of DC commitment to both cDCs and pDCs (27–29). Additional transcription factors such as Ikaros, PU.1, growth factor independent 1 transcriptional repressor (GFi1), interferon regulatory factor 8 (IRF8), basic leucine zipper ATF-like transcription factor 3 (BATF3), and inhibitor of DNA binding 2 (ID2) synergistically regulate DC development and subset specification through the engagement of different signaling pathways (30–35), as illustrated in **Figure 1**.

## Classification and Function of Dendritic Cell Subtypes

In humans, blood DC subtypes include CD11c^+^cDCs, that are CD1c^+^ or CD141^+^ cells, and CD11c^-^ pDCs, including CD123^+^ or CD303^+^ cells. Conventional DCs, previously termed type-1 (CD1c^+^) and type-2 (CD141^+^) myeloid DCs (mDCs), have recently reclassified as cDC2 and cDC1, respectively (36–38) (**Figure 1**). Conventional DCs exert a key function ranging from pathogen detection to cancer immunity as they are critical, through antigen presentation, to initiate specific T-cell responses. On the other, pDCs display high anti-viral activities due to their ability to produce type I interferon and are thought to be involved in immune tolerance (39, 40).

Finally, a new DC subtype is represented by the so-called monocyte-derived DCs (mo-DCs). Evidence shows that mo-DCs arise from monocytes recruited to the inflammatory site and express CD11c, CD1c, CD1a, Fc*ϵ*R1, IRF4, and ZBTB46. It is thought that mo-DCs promote CD4^+^ T cell polarization within inflammatory contexts (41). A synoptical view of the previous and actual classification of DCs is reported in **Table 1**.

**Table 1:** Synoptical classification of dendritic cell subsets.

Dendritic cell (DC) subtypes based on CD11c expression		Specific DC markers	Old classification	New classification
**Myeloid / Conventional**	CD11c^+^ DC	**CD1c / BDCA-1**	Type-1 Myeloid DC (mDC1)	**Conventional DC2 (cDC2)**
**Myeloid / Conventional**	CD11c^+^ DC	**CD141 / BDCA-3**	Type-2 Myeloid DC (mDC2)	**Conventional DC1 (cDC1)**
**Plasmacytoid**	CD11c^-^ DC	**CD123**	Plasmacytoid DC (pDC)	**Plasmacytoid DC (pDC)**
		**CD303 / BDCA-2**		
**Monocyte-derived DC**	CD11c^+^ DC	**CD1c**	–	**Monocyte-derived DC (mo-DC)**
		**CD1a**		
		**FcεR1**		
		**CD206**		

DC, dendritic cell; mDC, myeloid DC; cDC, conventional DC; Mo-DC, monocyte-derived DC; pDC, plasmacytoid DC; BDCA, Blood Dendritic Cell Antigen; FcεR1, Fc Fragment of IgE Receptor 1.

## Dendritic Cell Activation and Functional Maturation

Mature DCs display phenotypic and functional profiles distinct from their naïve (immature) counterparts. Immature DCs express low levels of major histocompatibility complex (MHC) and co-stimulatory molecules and are usually found in peripheral tissues where they play as sentinels for immune monitoring. These cells can endocytose and process antigens but are poorly effective in generating peptide-MHC complexes to ensure optimal antigen presentation and efficient T-cell activation (42–45). Tissue damage, inflammatory processes, microorganisms, and tumor-derived products may promote the maturation of DCs. After that, these cells lose endocytic activity, increase MHC-peptide complexes, up-regulate co-stimulatory molecules, and secrete inflammatory cytokines essential for the activation of T-cell responses (46, 47). Lastly, following maturation, DCs acquire an increased migratory potential that allows them to move into different compartments, such as non-lymphoid and lymphoid tissues and blood (48, 49).

## Dendritic Cell Subsets in the Human Lung Microenvironment

Due to their anatomy and function, the lungs are vital organs constantly exposed to the external environment. Consequently, inhaled particles of different nature and origin and potential pathogens need to be efficiently counteracted by a finely adjusted immune response to preserve lung health (50). The activity of lung DCs mainly depends on their organ distribution. For instance, DCs located in the alveolar septa have many dendritic projections able to continuously sample, while those located in the conducting airways seem to do so most rarely (51). Usually, DCs exist in an immature state in the lung periphery, skilled to take up inhaled particulate and soluble antigens. Upon activation, lung DCs, as previously described, become qualified to (52) induce a tailor-made immune response by T-cells (T-helper cell (Th) type 1, Th2, or Th17, depending on the type of pathogen) and B-cells (53, 54).

The lack of validated markers and technical difficulties in obtaining human lung tissues for investigation has significantly limited human lung DC subsets’ characterization and functional studies. Since the first observations by Demetds et al., who initially identified human lung DC subsets through the BDCA markers previously applied to characterize blood DCs (55), understanding pulmonary DC subtypes has improved chiefly only in the last few years. In particular, both genomic and functional studies have shown that human epithelial-associated DCs can be divided into four major subpopulations: pDCs, cDC2 CD1c^+^, cDC1 CD141^+,^ and mo-DCs (36–38, 41). More recently, lung DCs have been reclassified into five subtypes based on the differential expression of Langerin, CD1c, and CD14 (56). Interestingly, transcriptome analysis performed in bronchoalveolar lavage (BAL) samples has revealed in the human lower respiratory tract the existence of Langerin^+^, CD14^+,^ and CD14^−^ subsets of CD1c DCs functionally related with alveolar macrophages. Noteworthy, the higher mRNA expression levels of several dendritic cell-associated genes, including CD1, FLT3, CX3CR1, and CCR6, have disclosed a specific gene signature of DCs distinct from that of monocytes/macrophages (56). **Figure 1** synthetically depicts the DC subtype differentiation in the lung.

## The Role of DCs in IPF Pathogenesis

The involvement of DCs in the pathogenesis of IPF is a challenging field of relatively recent interest, with only a few reports available in humans.

In 2006 it was first reported that fully mature DCs expressing CD40, CD83, CD86, and DC-lysosome-associated membrane protein, along with non-proliferating B and T lymphocytes, contribute to the creation of ectopic organized lymphoid structures in the lung of IPF patients (57). Conversely, immature DC subsets seem to heavily infiltrate the IPF lungs, specifically in areas of epithelial hyperplasia and fibrosis, and to be present in the BAL fluid (58–60). It is thought that fibroblastic foci of IPF patients can orchestrate blood immature DC recruitment through chemokines’ expression (CCL19, CXCL12, and CCL21) (58, 61). This effect may maintain a condition of chronic inflammation by maturing DCs *in situ* within ectopic lymphoid follicles. Two physiologically relevant models showed that both human and mouse lung fibroblasts are critically involved in DC trafficking by secreting chemokines that play a crucial role in fibrosis and inflammation (62). Accordingly, co-cultures of DCs with lung fibroblasts from control subjects and IPF patients further confirmed the *in vitro* ability of lung fibroblasts to modulate the activation and maturation of DCs. These findings suggest that IPF fibroblasts might sustain chronic inflammation and immune responses by locally maintaining a pool of immature DCs (63). In a clinical trial published in 2015, the DC-specific growth factor Flt3L was found to increase cDC1 and cDC2 cell populations’ precursors in bone marrow biopsies and peripheral blood samples from healthy volunteers (64). Following this finding, Flt3L has further been shown to be up-regulated in the serum and lung tissue of IPF patients, likely contributing to the accumulation of lung DCs during pulmonary fibrogenesis (65).

**Figure 2:**
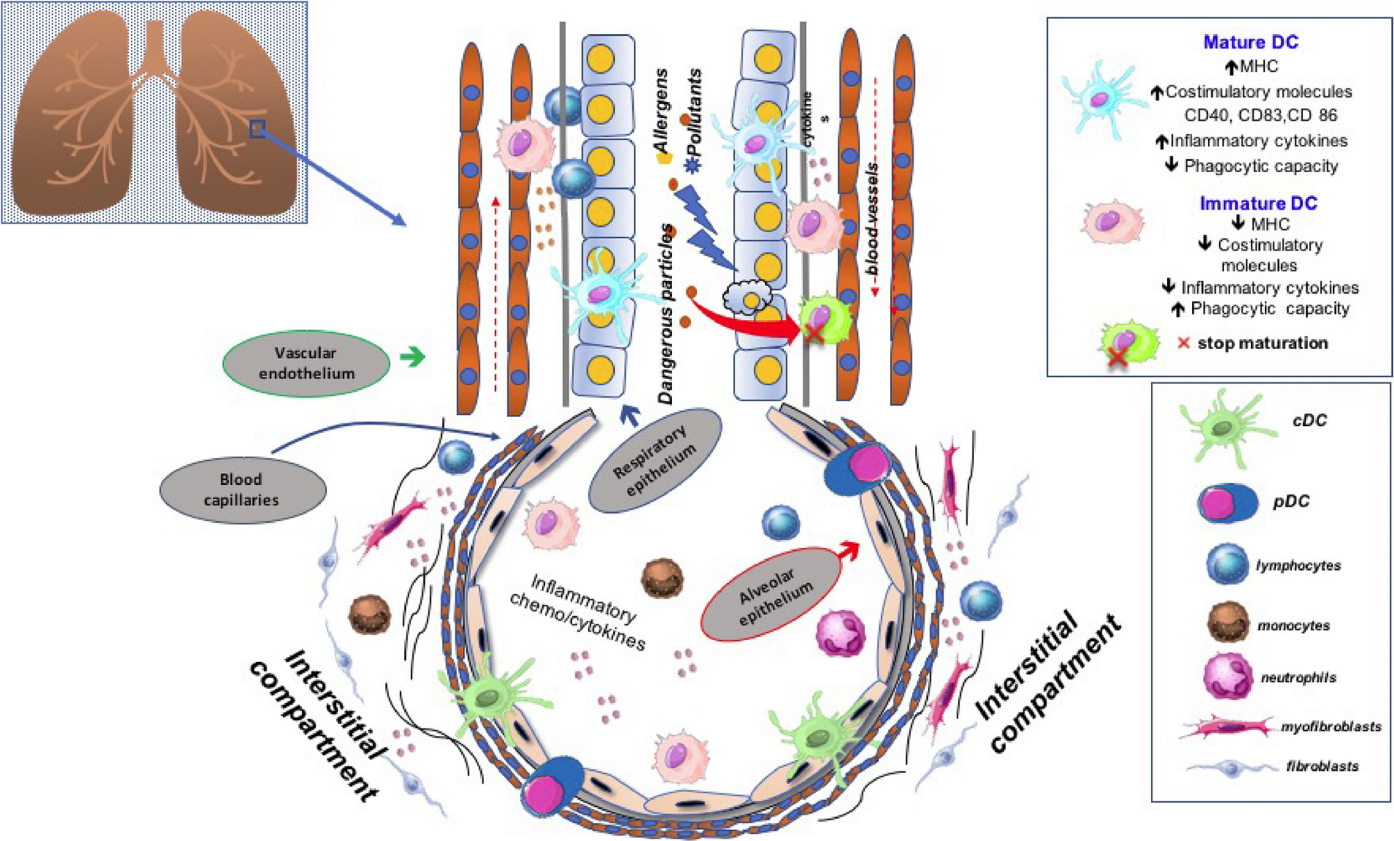
Dendritic cells (DC) are located in the lung interstitium and alveoli, where they act as sentinel cells. Any imbalance of their frequency distribution and functional status may have significant consequences in disease pathogenesis. Emerging evidence suggests that plenty of local factors along with different arrays of chemo-cytokines can modulate DCs maturation in the lung of patients affected by idiopathic pulmonary fibrosis, thus affecting their tolerogenic or immunogenic properties.

We previously showed that quantitative reduction of blood DCs was a feature shared by other respiratory diseases, including chronic obstructive pulmonary disease (COPD) and obstructive sleep apnea (66–68). We have recently also investigated the distribution of peripheral DCs subtypes in a prospective cohort of therapy naïve IPF patients. All blood DC subsets were severely depleted in the context of a pro-inflammatory milieu characterized by high expression levels of reactive oxygen species (ROS) and interleukin (IL)-6. In agreement with data previously reported, we likely attributed such a depletion, at least in part, to an increased cell turnover and recruitment at the lung level. Noteworthy, IL-6 levels and perturbations of the cDC2 subset were not influenced by anti-fibrotic therapies but were associated with reduced survival. Of note, low frequencies of cDC2 were an independent predictive biomarker of worse prognosis (69). **Figure 2** shows the role of DC subtypes undergoing the maturation process in the fibrotic lung tissue. Certainly, as mentioned, DCs involvement is not exclusive to IPF as it may also affect other respiratory diseases. In this context, it is worthy of note the report by Naessens T et al. The Authors have shown that cDC2 are potent inducers of T follicular helper cells and contribute to tertiary lymphoid tissue formation in the lung of COPD patients (70).

## The Way Forward: Similarities With Cancer Biology and Rationale for Immune-Targeted Therapies

In the light of the above evidence, DCs appear to play a role in the fibrotic process and, more specifically, in IPF pathogenesis. IPF notably shares many similarities with lung cancer, ranging from genetics to clinical behavior (71). It is also estimated that the overall cancer incidence in IPF patients is 29 cases per 1000 persons-years, with lung neoplasms being the most frequent ones (72). DC alterations have been widely studied and characterized in solid and blood malignancies (73). Like the liver fibrosis model leading to tumorigenesis (22), DC imbalance and functional impairment may represent a pathogenic bridge between IPF and cancer. This aspect merits further investigation for its prevention and therapeutic repercussions (13, 69). In this regard, DC-based treatments represent emerging alternatives to conventional chemotherapy in cancer patients (74), while such an approach is conceptually missing in fibrosis-related diseases. The lack of animal models that faithfully reproduce IPF pathogenesis is undoubtedly a significant limit in this setting. Despite this, the bleomycin model of inflammation-driven pulmonary fibrosis has still helped explore different purposes over time. In this regard, it has been shown that the immune-mediator VAG539 was able to attenuate the hallmarks of bleomycin-induced lung injury through the inactivation of DCs, suggesting a crucial role of these cells across the modulation of both inflammation and fibrosis (75). Likewise, infusion of CD11c-diphtheria toxin (DT) receptor (DTR) in bleomycin-treated mice prompted DCs depletion, thus mitigating lung fibrosis (76). Indeed, both studies have some limitations. First, the expression of aryl hydrocarbon receptor as the key molecular target of VAG539 is not restricted to DCs (77), and, second, the infusion of DT to CD11c-DTR mice depletes not only DCs but also pulmonary macrophages as CD11c is highly expressed on both cell types (78). Even with the awareness of these limitations, we believe that this area of interest deserves wider attention. Accordingly, recent clinical trials have explored the safety and efficacy of recombinant human Flt3L in healthy volunteers and cancer therapy to trigger DC expansion in humans (79–81). Interestingly, recombinant Flt3L increased the numbers of CD11b^+^ DCs, reducing lung fibrosis in wild-type (WT) mice exposed to AdTGF-beta1 (65).

IPF remains, for the most part, an unexplored field due to the non-recognition of the trigger cause. Perturbations of the lung microbiome and viral infections have been hypothesized to have a potential link with the development of IPF (82–86). Therefore, it is not negligible that any dysregulation of DCs, as major APCs and anti-viral effectors, may actively contribute to the puzzle of IPF pathogenesis through a wider involvement at different levels. Overall, accumulated evidence and related considerations further strengthen the concept that participation of DCs in the fibrotic process could be a driving force for future deepening.

## Conclusion

Interpreting the involvement of the immune response in the pathogenesis of IPF has become a prosperous field of investigation only in recent years. New reports reveal expanding potential pathogenic roles for DCs in lung fibrosis. These findings promise to open new scenarios to understand better the cause and the biological mechanisms underlying the disease. Further efforts and challenges will be to evaluate their potential in terms of easy to perform biomarkers predictive of clinical behavior and targets of immune-based treatments. In analogy with cancer, combination therapy strategies with anti-fibrotic drugs could optimistically represent a milestone shortly.

## Author Contributions

All authors contributed to the article and approved the submitted version.

## Conflict of Interest

The authors declare that the research was conducted in the absence of any commercial or financial relationships that could be construed as a potential conflict of interest.
